# *Qiliqiangxin* protects against anoxic injury in cardiac microvascular endothelial cells *via* NRG‐1/ErbB‐PI3K/Akt/mTOR pathway

**DOI:** 10.1111/jcmm.13111

**Published:** 2017-03-08

**Authors:** Jingfeng Wang, Jingmin Zhou, Yanyan Wang, Chunjie Yang, Mingqiang Fu, Jingjing Zhang, Xueting Han, Zhiming Li, Kai Hu, Junbo Ge

**Affiliations:** ^1^ Department of Cardiology Shanghai Institute of Cardiovascular Diseases Zhongshan Hospital Fudan University Shanghai China; ^2^ Department of Cardiology Shandong University Jinan Shandong China; ^3^ Department of Cardiology People's Hospital of Nanbu County Nanchong Sichuan China

**Keywords:** *Qiliqiangxin*, cardiac microvascular endothelial cell, neuregulin, anoxia, angiogenesis, apoptosis

## Abstract

Cardiac microvascular endothelial cells (CMECs) are important angiogenic components and are injured rapidly after cardiac ischaemia and anoxia. Cardioprotective effects of *Qiliqiangxin (QL)*, a traditional Chinese medicine, have been displayed recently. This study aims to investigate whether *QL* could protect CMECs against anoxic injury and to explore related signalling mechanisms. CMECs were successfully cultured from Sprague‐Dawley rats and exposed to anoxia for 12 hrs in the absence and presence of *QL*. Cell migration assay and capillary‐like tube formation assay on Matrigel were performed, and cell apoptosis was determined by TUNEL assay and caspase‐3 activity. Neuregulin‐1 (NRG‐1) siRNA and LY294002 were administrated to block NRG‐1/ErbB and PI3K/Akt signalling, respectively. As a result, anoxia inhibited cell migration, capillary‐like tube formation and angiogenesis, and increased cell apoptosis. *QL* significantly reversed these anoxia‐induced injuries and up‐regulated expressions of NRG‐1, phospho‐ErbB2, phospho‐ErbB4, phospho‐Akt, phospho‐mammalian target of rapamycin (mTOR), hypoxia‐inducible factor‐1α (HIF‐1α) and vascular endothelial growth factor (VEGF) in CMECs, while NRG‐1 knockdown abolished the protective effects of *QL* with suppressed NRG‐1, phospho‐ErbB2, phospho‐ErbB4, phospho‐Akt, phospho‐mTOR, HIF‐1α and VEGF expressions. Similarly, LY294002 interrupted the beneficial effects of *QL* with down‐regulated phospho‐Akt, phospho‐mTOR, HIF‐1α and VEGF expressions. However, it had no impact on NRG‐1/ErbB signalling. Our data indicated that *QL* could attenuate anoxia‐induced injuries in CMECs *via* NRG‐1/ErbB signalling which was most probably dependent on PI3K/Akt/mTOR pathway.

## Introduction

Angiogenesis, the formation of new blood vessels from pre‐existing vasculature involving the proliferation, migration, sprouting and tube formation of endothelial cells (ECs), is essential in the myocardial repair post‐infarction. CMECs, a specific cell type derived from coronary microvessels, are quite different from macrovascular ECs because of their capacity of rapid spreading and fast tube formation and their ability to promote angiogenesis 1, 2. Removal of these CMECs has been shown to have significant detrimental effects on the contractile function of the adjacent cardiac myocytes 3. It is already established that both acute and chronic hypoxia exert an inhibitory effect on cultured CMEC proliferation 4. Ischaemia/reperfusion injury which induces the release of soluble pro‐apoptotic mediators from ECs to surrounding cardiac myocytes may even promote myocyte apoptosis 5. Hence, it is of great importance to protect CMECs from anoxic injuries.

The neuregulins (NRGs), a family of ligands for ErbB receptors, were first discovered based on their ability to stimulate the growth of cultured cells from the central nervous system. Of the four genes known to encode NRG, only NRG‐1 and NRG‐2 isoforms have been shown to be expressed in the heart, while only NRG‐1 Type I isoforms continue to be expressed in the endocardium and coronary microvasculature of adult heart 6. NRG‐1 was found in a variety of ECs including: cultured human umbilical vein EC (HUVEC), human coronary artery and endocardial endothelium, and coronary microvascular EC from adult rat ventricle 7, 8. Receptors for the cardiac‐specific NRGs include ErbB2, ErbB3 and ErbB4. Both ErbB2 and ErbB4 are expressed in the pre‐ and post‐natal heart, while binding of NRG‐1 to ErbB4 induces phosphorylation of itself and its co‐receptor ErbB2. NRG‐1/ErbB signalling has been shown to play an important role in paracrine and autocrine signalling in heart, regulating both myocyte and EC function, including anti‐apoptosis and angiogenic responses 8, 9, 10, 11. Previous studies showed that overexpression of NRG‐1 had cardioprotective effects against ischaemic injury through PI3K/Akt‐dependent mechanism in cultured cardiomyocytes 12, 13, 14.

*QL*, a specific traditional Chinese medicine extracted from 11 distinct herbs including *Radix astragali, Aconite Root, Ginseng, Salvia miltiorrhiza, Semen lepidii apetali, Cortex Periplocae Sepii Radicis, Rhizoma Alismatis, Carthamus tinctorius, Polygonatum Odorati, Seasoned Orange Peel* and *Rumulus Ginnamomi,* has been reported to inhibit cardiac remodelling and cardiomyocyte apoptosis after myocardial infarction 15, 16. A multicenter randomized double‐blind parallel‐group placebo‐controlled study demonstrated that 12 weeks of *QL* treatment reduced the levels of N‐terminal pro‐B‐type natriuretic peptide (NT‐pro‐BNP) in 512 chronic heart failure patients 17. We previously observed the beneficial effects of *QL* in that *QL* promoted angiogenesis and reduced apoptosis in rats with experimental myocardial infarction, and these effects were linked with NRG‐1 up‐regulation and PI3K/Akt activation 18. However, whether these protective effects of *QL* are NRG‐1/ErbB or PI3K/Akt‐dependent remains unclear. The present study aims to assess the effects of *QL* on CMECs under anoxic condition and to explore the potential mechanisms.

## Materials and methods

### Materials and reagents

*QL* superfine powder was provided by Shijiazhuang Yiling Pharmaceutical Corporation (Shijiazhuang, China). Low‐glucose Dulbecco's modified Eagle's medium (DMEM), foetal bovine serum (FBS) and trypsin were purchased from Life Technologies (Carlsbad, CA, USA). Hypoxia incubator chamber was purchased from StemCell Technologies (Vancouver, BC, Canada). NRG‐1 siRNA and siPORT™ NeoFX™ Transfection Agent were purchased from Shanghai GenePharma Co., Ltd. (Shanghai, China) and Life Technologies, respectively. Matrigel was purchased from BD Biosciences (Billerica, MA, USA) and wound healing culture inserts from *ibidi* (Munich, Germany). In Situ Cell Death Detection Kit was purchased from Roche (Penzberg, Germany) and Caspase‐3 activity assay kit from Beyotime Institute of Biotechnology (Haimen, China). Rat NRG‐1 ELISA Kit was obtained from Cloud‐Clone Corp (Houston, TX, USA). Primary antibodies for factor VIII (sc‐14014), NRG‐1 (sc‐348) and VEGF (sc‐152) were purchased from Santa Cruz (Santa Cruz, CA, USA); antibodies for phospho‐ErbB2 (ab47262), phospho‐ErbB4 (ab61059) and peroxisome proliferator‐activated receptor‐γ (PPARγ) (ab209350) were from Abcam (Cambridge, UK); antibodies for pan‐Akt (#4685), phospho‐Akt (Ser473) (#4058), phospho‐mammalian target of rapamycin (mTOR) (Ser2448) (#2971), hydroxy‐HIF‐1α (Pro564) (#3434) and LY294002 (#9901) were obtained from Cell Signaling Technology (Boston, MA, USA). PrimeScript™ RT Reagent Kit and SYBR^®^ Premix Ex Taq™ Kit for RT‐PCR were purchased from Takara (Shiga, Japan).

### Preparation of *QL*

The origin, harvest time, medicinal composites and processing technology of the 11 pharmaceutical ingredients of *QL* were strictly normalized and standardized, which was available online at https://doi.org/10.1155/2013/378298 19. *QL* extract, in the form of dried superfine powder (≤10 μm), was concocted by boiling, steaming and drying and was seriously authenticated and standardized on the basis of marker compounds to achieve quality control according to the Chinese Pharmacopoeia 2005 (National Pharmacopoeia Committee, 2005). In the present *in vitro* study, *QL* superfine powder was dissolved in DMEM at the concentration of 10 mg/ml and filtrated by a 0.22‐μm micropore filter, which was recommended by Shijiazhuang Yiling Pharmaceutical Corporation. The supernatant was then collected and stored at 4°C.

### Isolation and identification of CMECs

Two‐week‐old Sprague‐Dawley male rats were euthanized by cervical dislocation after anaesthesia with 5% isoflurane and were placed in 70% ethanol for 5 min. Then, the hearts were rapidly excised after thoracotomy and rinsed with ice‐cold phosphate‐buffered saline (PBS) solution under sterile conditions. Explant cultivation was applied to isolate CMEC 1. After modifications by cutting off atria and great vessels, the outer and inner layer of left ventricle was dissected away to remove epicardial arteries, larger penetrating vessels and endocardial ECs. The remaining left ventricular tissues were minced into 1‐mm^3^ pieces and uniformly plated on 10‐cm culture dishes, which were humidified with 1 ml 10% FBS in advance. Tissue pieces were then cultured in a humidified incubator with 5% CO_2_–95% air for 4 hrs. Once the pieces were firmly attached to culture dishes, 6 ml DMEM supplemented with 10% FBS was added to each dish and kept in 5% CO_2_–95% air incubator for another 68 hrs until abundant polygonal or star‐like cells crept out of myocardial tissues. After removal of tissue pieces and non‐adherent cells by careful washing, 6 ml DMEM was again added to each dish for further culture. When the cells were 80–90% confluent, they were washed with PBS, digested in 0.1% trypsin and passaged onto 6‐well plates, followed by incubation at 37°C for approximately 12 hrs allowing cells to adhere and spread on the substrate. CMECs were identified by morphological characteristics, including uniform ‘cobblestone’ morphology, and positively immunostained with anti‐factor VIII antibody. For immunofluorescence assay, cells were washed with PBS and fixed in 4% paraformaldehyde for 10 min. After being sealed with 5% bovine serum albumin for 60 min., cells were incubated over night with anti‐factor VIII antibody (dilution 1:50) at 4°C. Cells were then washed again with PBS and incubated 1 hr with Alexa Fluor^®^ 555‐conjugated secondary antibodies (A‐21428; Invitrogen, Rockford, IL, USA; dilution 1:200). Finally, DAPI (Sigma‐Aldrich, St. Louis, MO, USA) was added for 10 min. and cells were visualized under fluorescence microscope. Three independent experiments were carried out for CMEC identification.

### Screening for optimal anoxia time

Hypoxia‐inducible factor‐1 (HIF‐1) consists of a constitutively expressed subunit β and an oxygen‐regulated subunit α. Under normally oxygenated conditions, HIF‐1α levels are degraded substantially by a family of prolyl hydroxylases. While under anoxic conditions, the prolyl hydroxylases cease to function properly and HIF‐1α increases and becomes stable 20. So in the present study, we carried out screening for optimal anoxia time in light of HIF‐1α expression. CMECs were placed in a hypoxia cultivator containing a gaseous mixture of 95% N_2_ and 5% CO_2_ at 37°C for durations of 0, 3, 6, 12 and 24 hrs, respectively. Subsequently, proteins of CMECs were extracted with RIPA lysis buffer (Beyotime, China) and subjected to Western blot analysis for HIF‐1α expression. That with the highest HIF‐1α expression was considered as optimal anoxic exposure time.

### *In vitro* small‐interfering RNA technique

Scrambled siRNA as a negative control and three specific siRNAs targeting NRG‐1 (sequences displayed in Table 1) together with siPORT™ NeoFX™ Transfection Agent were used for siRNA experiments. When CMECs reached 70–75% confluence, the media were aspirated and cells were resuspended in DMEM. Diluted siRNA and diluted siPORT™ NeoFX™ Transfection Agent were mixed and incubated before being dispensed into 6‐well plate. Cell suspensions were then overlaid onto the transfection complexes at about 2 × 10^5^ cells/well. The final siRNA (either negative control siRNA or NRG‐1 siRNAs) concentration was 5 nmol/l. For control group, only siPORT™ NeoFX™ Transfection Agent diluted in Opti‐MEM^®^ I medium was administrated. Cells were then cultured at 37°C for 24 hrs when the medium was replaced with fresh DMEM. After 24 hrs of siRNA transfection, NRG‐1 expression in CMECs was evaluated by Western blot to confirm successful inhibition of NRG‐1 expression.

**Table 1:** Sequences for negative control siRNA and siRNAs against NRG‐1

siRNA	Sequences	
	Sense (5ʼ–3ʼ)	Antisense (5ʼ–3ʼ)
Negative control	UUCUCCGAACGUGUCACGUTT	ACGUGACACGUUCGGAGAATT
NRG‐1 siRNA‐1726	GGUGAAUCAAUACGUAUCUTT	AGAUACGUAUUGAUUCACCTT
NRG‐1 siRNA‐1883	GAGAGCAUCAUUUCAGAAATT	UUUCUGAAAUGAUGCUCUCTT
NRG‐1 siRNA‐2253	GGCUACGGGAGAAGAAAUATT	UAUUUCUUCUCCCGUAGCCTT

### Experimental protocol

Subcultured rat CMECs of passage 2 at approximately 70–80% confluency were used in subsequent experiments. After preparation of siRNA‐transfected CMECs, anoxia was achieved by placing CMECs into a hypoxia incubator chamber (95% N_2_/5% CO_2_, v/v) for 12 hrs. To further investigate PI3K/Akt pathway in NRG‐1/ErbB signalling, the PI3K/Akt inhibitor LY294002 was also adopted. In our previous study, trypan blue staining elicited a highest CMEC viability (64.5 ± 2.1%) at 0.5 mg/ml *QL* treatment. Thus, in the present study, cells (either NRG‐1 siRNA transfected or non‐transfected) were treated with *QL* (0.5 mg/ml) and/or LY294002 (50 μmol/l) 1 hr before anoxia exposure. The control group was maintained in a humidified atmosphere of 5% CO_2_ at 37°C till end of study.

### Cell migration assay

To investigate the effect of *QL* on cell migration ability, *ibidi* culture inserts were used to simulate ‘scratch‐wound healing’ assay. NRG‐1 siRNA transfection was conducted in advance. Cell suspensions were prepared at 5 × 10^5^ cells/ml in DMEM. *Ibidi* culture inserts consisting of two reservoirs separated by a 500‐μm‐thick wall were planted onto 35‐mm culture dishes using sterile tweezers. Seventy microlitre cell suspensions were then added into the two reservoirs each and incubated at 37°C/5% CO_2_. When the cells were 100% confluent round the insert, it was gently removed creating a gap of 500 μm. DMEM was then added into culture dishes up to 2 ml for each, followed by subsequent interventions, including *QL* and/or LY294002 treatment. After 12 hrs of anoxia, migration was photographed under an inverted‐phase contrast microscope along the cell‐free zone (100×). Cell migration was assessed using Wimasis WimScratch software, measuring the percentage of scratch area which was not covered by cells at three different sites and comparing results to 0‐hr time‐point. A wider scratch area suggested a poor migration ability, and vice versa.

### Capillary‐like tube formation assay on Matrigel

*In vitro* EC tube formation assay on Matrigel culture was used as a surrogate assay for angiogenic potential. Matrigel (150 μl) was thawed at 4°C and paved on a 24‐well plate for solidification at 37°C for 30 min. CMECs, either siRNA transfected or non‐transfected, were trypsinized, resuspended and treated with *QL* and/or LY294002 at final concentration of experiment 1 hr before being seeded onto the Matrigel plates at 1 × 10^5^ cells/well in triplicate. After 12 hrs of anoxia, the tubular formation of CMECs was observed and photographed using an inverted‐phase contrast microscope in three random fields (50×). Image analysis was carried out by Wimasis WimTube software to deliver tubule and net characteristics including total number of tubules, loops and branch points.

### Cell apoptosis assay

Cell apoptosis was detected by TUNEL assay using *In Situ* Cell Death Detection Kit and caspase‐3 activity using Caspase‐3 activity assay kit according to the manufacture's instructions. For TUNEL staining, CMECs were cultured on coverslips on a 24‐well plate in advance. After intervention, cells were successively fixed in 4% paraformaldehyde for 1 hr, blocked with 3% H_2_O_2_ in methanol for 10 min. and incubated in a permeabilization solution (0.1% Triton X‐100) for 2 min. on ice. Subsequently, 50 μl aliquots of the TUNEL reaction mixture were added on each sample, followed by incubation for 1 hr at 37°C in the dark. After being rinsed with PBS three times, cells were counterstained with DAPI for 10 min. Finally, TUNEL‐positive nuclei were visualized by fluorescence microscopy (Leica, Germany) and counted from three randomly chosen fields (200×) in each sample. Data were expressed as ratio of TUNEL‐positive nuclei to total nuclei based on DAPI‐positive nuclear staining. Caspase‐3 activity was measured with a Caspase 3 Activity Assay Kit. Briefly, cells were lysed and the supernatants were collected after treatment. Supernatants containing 200 μg of protein were then incubated with 10 μl of the caspase‐3 substrate acetyl‐Asp‐Glu‐Val‐Asp‐*p*‐nitroanilide (Ac‐DEVD‐*p*NA) at 37°C for 2 hrs in the dark. The release of *p*‐nitroaniline (*p*NA) was quantified by measuring the absorbance with a microplate reader at 405 nm. Caspase‐3 activity was expressed as the fold changes compared with the control value.

### Enzyme‐linked immunosorbent assay (ELISA) for NRG‐1 expression

After the media being collected and centrifuged, levels of NRG‐1 secreted into the cell culture supernatant were measured with commercially available ELISA kit according to the manufacturer's instructions. Standard or sample, detection reagent, substrate solution and stop solution were sequentially added to wells and incubated with repeated washing as appropriate. Optical density at 450 nm was immediately measured with a plate reader, and sample values were then calculated from the standard curve.

### Western blot analysis

After 12 hrs of anoxia, cells were lysed in RIPA lysis buffer (Beyotime, China) supplemented with 1 mmol/l PMSF and phosphates inhibitor (Pierce, Rockford, IL, USA) on ice bath. Lysates were then centrifuged at 12,000 × *g* for 10 min. to remove the cell debris. After protein concentration was determined with a BCA protein assay kit (Beyotime, Nantong, China), aliquots of protein (20 μg) were electrophoresed on 12% SDS‐PAGE gels and then transferred onto polyvinylidene difluoride membranes (Thermo Scientific, Waltham, MA, USA). After being blocked with 5% non‐fat dry milk for 1 hr, membranes were probed with primary antibodies against NRG‐1 (dilution 1:500), phospho‐ErbB2 (dilution 1:500), phospho‐ErbB4 (dilution 1:500), pan‐Akt (dilution 1:1000), phospho‐Akt (Ser473) (dilution 1:1000), phospho‐mTOR (Ser 2448) (dilution 1:1000), PPARγ (1:500), hydroxy‐HIF‐1α (Pro564) (dilution 1:1000) and VEGF (dilution 1:500) at 4°C overnight. Membranes were then washed with TBST and further incubated with appropriate horseradish peroxidase‐conjugated secondary antibodies (#A21020; Abbkine, Inc., Redlands, CA, USA) (dilution 1:5000) at 37°C for 2 hrs. Finally, immunocomplexes were visualized by ECL Plus (Thermo Scientific) and the relative expression levels were normalized to a loading control of GAPDH (MB001H; Bioworld Technology, Inc., St. Louis Park, MN, USA) (dilution 1:10,000).

### Real‐time quantitative reverse transcription‐polymerase chain reaction (RT‐qPCR)

Total RNA was first extracted from CMECs using TRIzol reagent according to the manufacturer's protocol (Invitrogen). RNA (500 ng) was then reverse‐transcribed into cDNA in a 10 μl reaction volume with PrimeScript™ RT Reagent Kit. RT‐qPCR was performed on an Applied Biosystems^®^ 7500 Real‐Time PCR Detection System (Life Technologies) with SYBR^®^ Premix Ex Taq™ Kit in a 20 μl reaction volume. RT‐PCR cycling included a denaturing step at 95°C for 30 sec., followed by 40 cycles of annealing step at 95°C for 5 sec. and extension at 60°C for 34 sec. with each sample analysed in triplicate. The primers used for PCR were as follows: NRG‐1, forward, CTTCGGTCAGAACGGAGCAA, reverse, ACAGTCGTGGAGTGATGGGC; HIF‐1α, forward, CCCTACTATGTCGCTTTCTTGG, reverse, TTTCTGCTGCCTTGTATGGGAG; GAPDH, forward, AGTGCCAGCCTCGTCTCATAG, reverse, CGTTGAACTTGCCGTGGGTAG. Relative mRNA expression was normalized to GAPDH as endogenous control by the standard 2^−ΔΔCt^ method.

### Statistical analysis

All assays were carried out in five independent experiments, and results were expressed as mean ± S.D. Differences between groups were determined by one‐way anova with the SNK *post hoc* test, and *P* < 0.05 was considered statistically significant. All analyses were carried out with SPSS 19.0 software (SPSS Inc., Chicago, IL, USA).

## Results

### CMEC was successfully isolated and positively identified

Primary isolated rat CMECs appeared as fusiform or polygonal cells around tissue granules after 48 hrs of incubation. After being cultured for 96 hrs, primary isolates reached approximately 90–100% confluency and demonstrated a ‘cobblestone’ appearance. Nearly all cells were positively immunostained with microvascular endothelium‐specific anti‐factor VIII antibody (Fig. S1A). Considering preceding evulsion of epicardial arteries, these factor VIII‐positive cells could be identified as CMECs. Of the incremental durations of anoxia time (0, 3, 6, 12, 24 hrs), HIF‐1α reached its peak level at 12 hrs of anoxia, suggesting a steady anoxic condition (Fig. S1B). Hence, we chose 12 hrs as optimal anoxia time‐point in the following study.

### Successful knockdown of NRG‐1 by small‐interfering RNA in cultured CMECs

Three siRNAs targeting NRG‐1(siRNA‐1726, 1883, 2253) were designed by GenePharma designer 3.0 programme and synthesized by Shanghai GenePharma Cooperation. Twenty‐four hours after siRNA transfection, we validated the efficiency of siRNAs against their targeted protein expression by immunoblotting. As a result, all three siRNAs reduced NRG‐1 expression in comparison with non‐transfected or negative‐transfected condition, among which siRNA‐1883 had the most significantly inhibitory effect on NRG‐1 expression by 53% (*P* < 0.01), a result corroborating the efficiency of transfection (Fig. S1C). So we chose NRG‐1 siRNA‐1883 as transfection siRNA in our subsequent experiments.

### *QL* promoted migration and angiogenesis and inhibited apoptosis in CMECs exposed to anoxia

**Figure 1:**
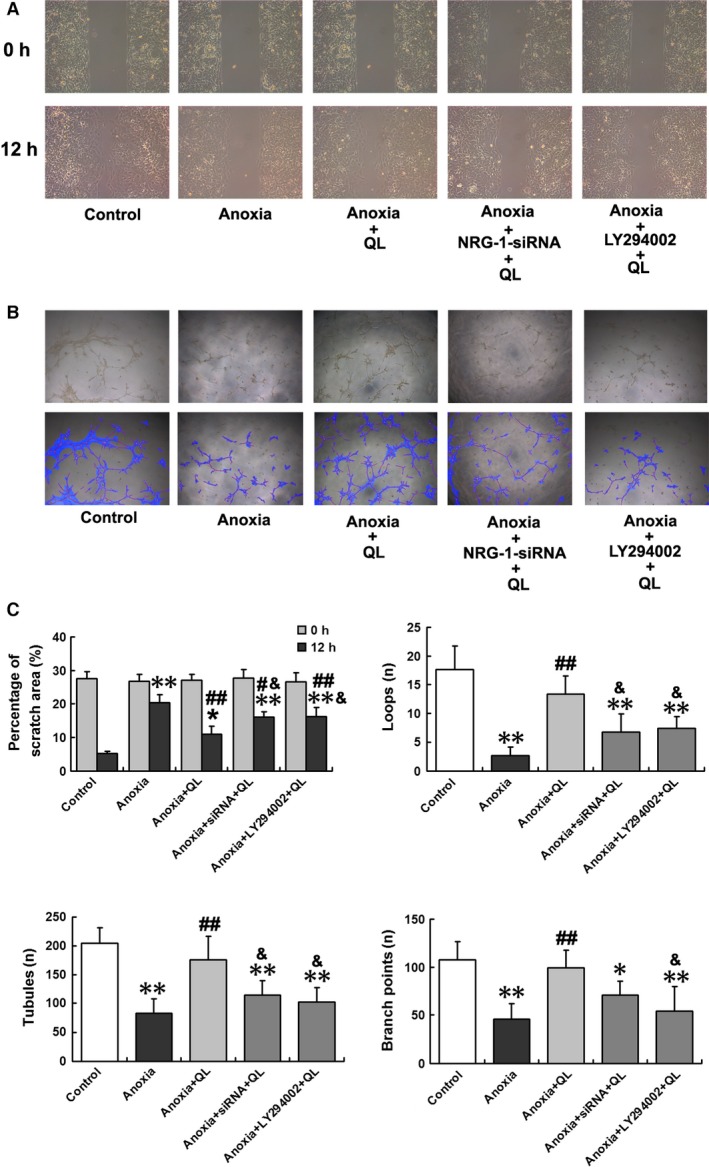
*QL* promoted CMECs migration and capillary‐like tube formation, which were abolished by NRG‐1 siRNA and LY294002 administration. Three random fields from each well were assessed. (**A**) Representative inverted‐phase contrast microscope images (100×) of cell migration and invasion of the scratch among different groups, as exhibited by percentage of scratch areas, which were quantified using Wimasis WimScratch software from 0 to 12 hrs of anoxia. (**B**) Tube networks including total number of loops, tubules and branch points were analysed under inverted‐phase contrast microscope (50×) at the end of experiment. Furthermore, a detailed overlay images were provided using Wimasis WimTube software, in which cell‐covered area was in blue, tubules in red, branch points in white and loops by yellow mark. (**C**) Bar graphs for percentage of scratch area and counts of loops, tubules, branch points among different groups. Percentage of scratch area was counted as scratch area divided by the total area in the same field. Capability of capillary‐like tube formation was described as total number of loops, tubules and branch points. Compared with control group, *P < 0.05, **P < 0.01; compared with anoxia group, ^#^ *P* < 0.05, ^#^^#^ *P* < 0.01; compared with anoxia+*QL* group, ^&^ *P* < 0.05.

Compared with normoxia condition, anoxic injury markedly impaired CMEC migration, as evidenced by a wider scratch area after 12‐hrs exposure to anoxia (20.33 ± 2.47% *versus* 5.33 ± 0.55%, *P* < 0.01). *QL*, however, increased CMEC movement to the wounded area under anoxic condition, presented as a relatively narrower scratch area (10.90 ± 2.36% *versus* 20.33 ± 2.47%, *P* < 0.01; Fig. 1A and C). Meanwhile, we also assessed capillary‐like tube formation on Matrigel as a model of *in vitro* angiogenesis. Total number of tubules, loops and branch points were compared among different intervention groups. Anoxia severely suppressed CMEC tube formation, as exhibited by reduced number of tubules, loops and branch points, while pre‐treatment with *QL* for 1 hr restored parameters of tube network formation in CMECs exposed to anoxia to near control (normoxia) level (Fig. 1B and C).

**Figure 2:**
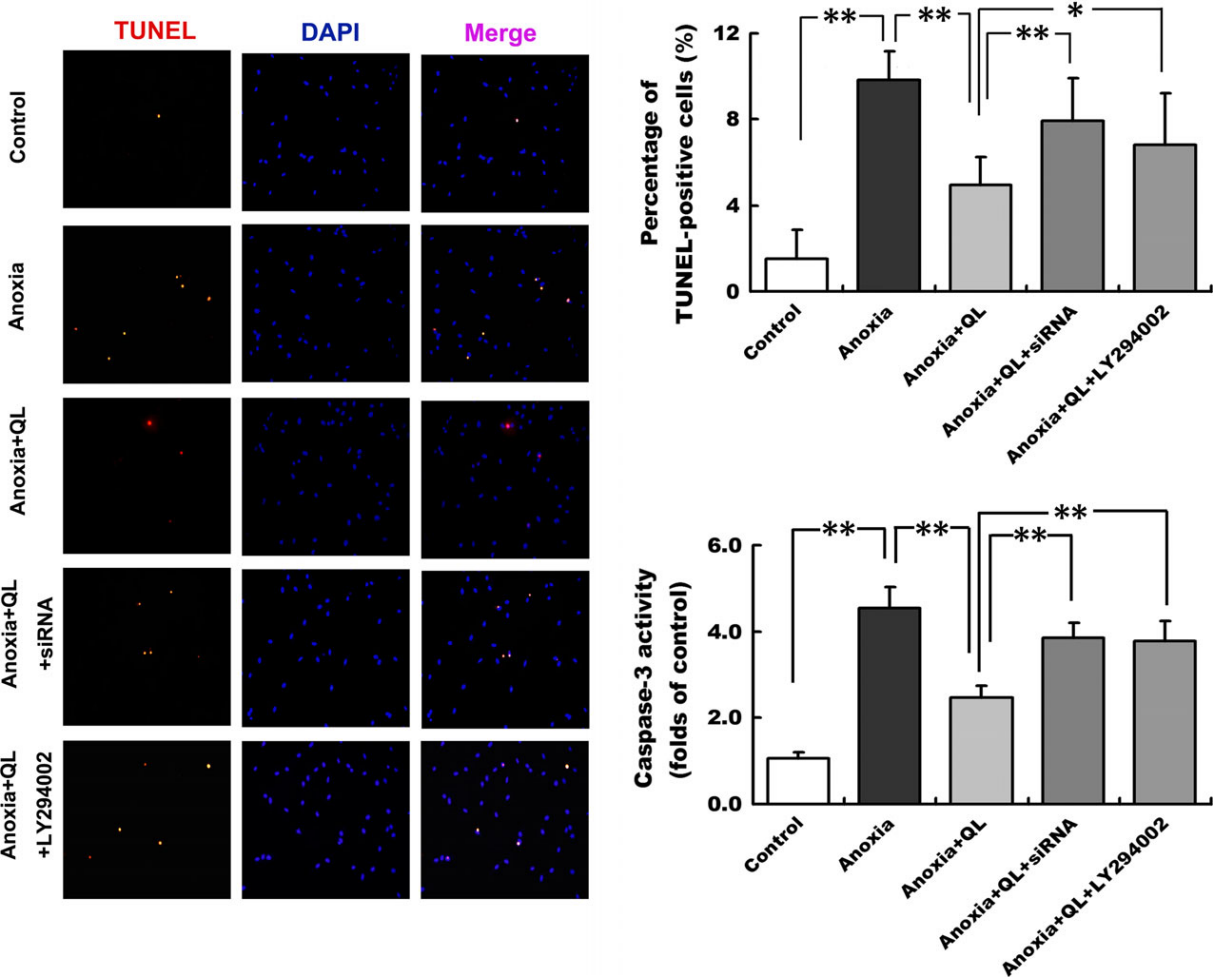
*QL* significantly reduced the percentage of TUNEL‐positive cells and caspase‐3 activity in CMECs exposed to anoxia. Both NRG‐1 siRNA and LY294002 attenuated such anti‐apoptotic effect. Representative photographs of double staining of TUNEL and DAPI at 12 hrs of anoxia were displayed. Red indicates TUNEL‐positive nuclei; blue indicates DAPI signals. Quantification of CMECs apoptosis was expressed as ratio of TUNEL‐positive nuclei to total nuclei in each randomly chosen field(200×). Caspase‐3 activity was also detected and expressed as fold changes compared with the control value. *P < 0.05, **P < 0.01.

Our previous study has demonstrated that *QL* attenuated cardiomyocyte apoptosis in rat model of post‐infarction heart failure 18. In the present study, we attempted to study whether *QL* played an anti‐apoptotic role in CMECs exposed to anoxia. In consequence, anoxic insult obviously increased percentage of TUNEL‐positive apoptotic cells (9.80 ± 1.38% *versus* 1.54 ± 1.34%, *P* < 0.01) and enhanced caspase‐3 activity, which was significantly declined in *QL*‐treated cells (4.98 ± 1.28% *versus* 9.80 ± 1.38%, *P* < 0.01; Fig. 2), indicating that *QL* protected CMECs from anoxic attack by increasing CMEC survival and inhibiting apoptosis.

### NRG‐1 knockdown and LY294002 attenuated the protective effects of *QL* on CMECs under anoxic condition

To explore whether NRG‐1/ErbB and PI3K/Akt signalling mediate the protective effects of *QL*, NRG‐1 siRNA and LY294002 were employed to inhibit NRG‐1 expression and Akt phosphorylation. *QL* enhanced migration and capillary‐like tube formation of CMECs exposed to anoxia, which was abolished by NRG‐1 siRNA transfection to a great extent, as exhibited by a wider scratch area (16.07 ± 2.46% *versus* 10.90 ± 2.36%, *P* < 0.05) and fewer tubules and loops on Matrigel (Fig. 1). Concomitantly, the anti‐apoptotic effect of *QL* was also weakened in response to knockdown of NRG‐1, as evidenced by a higher percentage of TUNEL‐positive cells (7.92 ± 1.99% *versus* 4.98 ± 1.28%, *P* < 0.01) and increased caspase‐3 activity, compared with NRG‐1 wild‐type cells (Fig. 2). Similarly, administration of LY294002 attenuated the beneficial effects of *QL* on anoxia‐induced CMEC injuries including lower migration (scratch area 16.17 ± 2.64% *versus* 10.90 ± 2.36%, *P* < 0.05), decreased capillary‐like tube formation and aggravating cell apoptosis (percentage of TUNEL‐positive cells 6.81 ± 2.41% *versus* 4.98 ± 1.28%, *P* < 0.05; Figs 1, 2).

### *QL* protected CMECs from anoxic injury in a NRG‐1/ErbB‐PI3K/Akt/mTOR‐dependent manner

**Figure 3:**
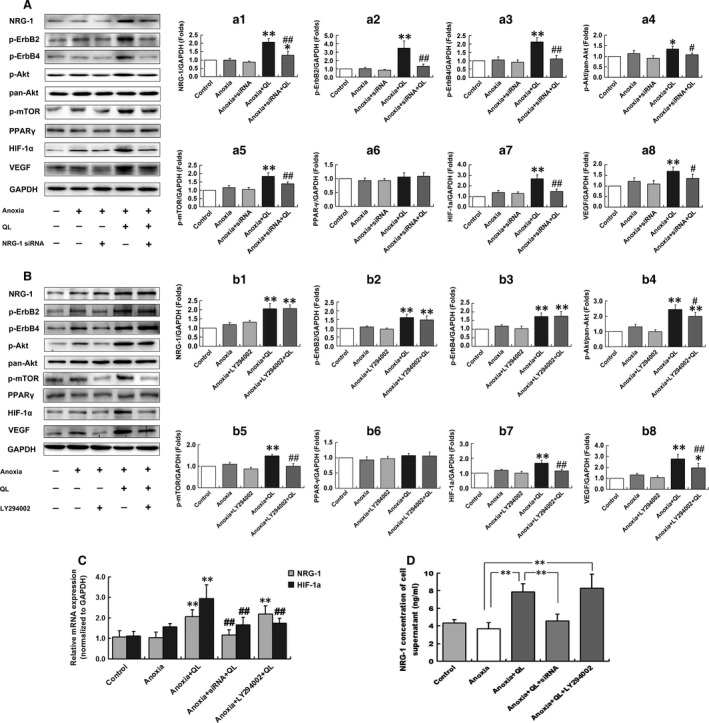
*QL* up‐regulated expressions of NRG‐1, phospho‐ErbB2, phospho‐ErbB4, phospho‐Akt, phospho‐mTOR, HIF‐1α and VEGF in CMECs exposed to anoxia. NRG‐1 siRNA transfection abolished these up‐regulations. LY294002 also interrupted up‐regulation of phospho‐Akt, phospho‐mTOR, HIF‐1α and VEGF by *QL*, but did not interfere with NRG‐1 and ErbB expressions. PPARγ expression was not regulated by *QL*. (**A**) Western blotting analyses for NRG‐1, phospho‐ErbB2, phospho‐ErbB4, phospho‐Akt, phospho‐mTOR, PPARγ, HIF‐1α and VEGF expressions in CMECs subjected to anoxia, *QL* treatment and NRG‐1 siRNA transfection. a1–a8 showed corresponding bar graphs. GAPDH and total Akt expressions served as internal control. (**B**) Western blotting analyses for NRG‐1, phospho‐ErbB2, phospho‐ErbB4, phospho‐Akt, phospho‐mTOR, PPARγ, HIF‐1α and VEGF expressions in CMECs subjected to anoxia, *QL* treatment and LY294002. b1–b8 showed corresponding bar graphs. GAPDH and total Akt expressions served as internal control. Representative blots from five independent experiments were displayed for (**A**) and (**B**). Compared with anoxia group, *P < 0.05, **P < 0.01; compared with anoxia+*QL* group, ^#^ *P* < 0.05, ^#^^#^ *P* < 0.01. (**C**) Real‐time quantitative PCR analyses for NRG‐1 and HIF‐1α mRNA expression normalized to GAPDH. Relative values were calculated for the ratio of NRG‐1 or HIF‐1α to GAPDH and expressed as folds of control. Compared with anoxia group, **P < 0.01; compared with anoxia+*QL* group, ^#^^#^ *P* < 0.01. (**D**) NRG‐1 concentration in cell supernatants detected by ELISA. *QL* promoted NRG‐1 secretion into extracellular space, which was abolished by NRG‐1 siRNA, but not by LY294002. **P < 0.01.

It is well known that CMECs are injured immediately after cardiac ischaemia and anoxia, preceding cardiomyocytes 5. The present study showed that *QL* up‐regulated HIF‐1α at both protein and mRNA levels, together with its downstream VEGF expression. However, in NRG‐1 knockdown CMECs, *QL* failed to up‐regulate HIF‐1α and VEGF expression. Likewise, LY294002 abolished this up‐regulation action. These results suggested that NRG‐1/ErbB and PI3K/Akt signalling was involved in the pro‐angiogenic effect of *QL* in anoxia‐induced injury *via* HIF‐1α and VEGF. To further interpret how these signalling pathways worked, we elicited that *QL* significantly up‐regulated NRG‐1 expression, ErbB2, ErbB4, Akt and mTOR phosphorylation in CMECs suffering from anoxia‐induced injury, but was incapable of activating ErbBs, Akt and mTOR in NRG‐1 knockdown CMECs. Nevertheless, the PI3K/Akt inhibitor, LY294002, abrogated *QL*‐induced phosphorylation of Akt and mTOR but did not suppress *QL*‐induced up‐regulation of NRG‐1/ErbBs. The protein expressions of PPARγ showed no significant difference among our experimental groups, indicating that PPARγ was not involved in regulation of CMECs function exposed to anoxia or on *QL* treatment (Fig. 3A, B and C). Considering NRG‐1 works mainly by being secreted into extracellular space and binding ErbB receptors, we evaluated NRG‐1 expression in cell supernatant by ELISA. *QL* significantly elevated supernatant NRG‐1 concentration under anoxic condition. Such elevation was suppressed by NRG‐1 siRNA transfection, but not by LY294002 (Fig. 3D). All these observations illustrated that NRG‐1/ErbB signalling mediated the protective effects of *QL* on CMECs subjected to anoxic injury, which was most likely to be PI3K/Akt/mTOR dependent.

## Discussion

The major findings of the present study can be summarized as follows: (*i*) *QL* protected CMECs against anoxic injuries through promoting angiogenesis and inhibiting apoptosis; (*ii*) NRG‐1 knockdown interrupted PI3K/Akt/mTOR pathway and attenuated the beneficial effects of *QL* on CMECs exposed to anoxia; (*iii*) these beneficial effects were mediated through PI3K/Akt/mTOR‐dependent NRG‐1/ErbB signalling pathway.

Ischaemic heart disease is a major cause of mortality and morbidity worldwide. Even with early coronary reperfusion post‐ischaemic insult, a decreased microvascular perfusion and chronically impaired cardiac function might still occur because of microvascular damage 21. *QL*, a Chinese compound medicine, was shown to be effective in heart failure following either myocardial infarction or pressure overload 15, 16, 22. Our study displayed the pro‐angiogenic and anti‐apoptotic effects of *QL* on CMECs. The potential mechanism involved may be associated with NRG‐1/ErbB signalling that depended on PI3K/Akt/mTOR pathway.

Several active ingredients of this *QL* extract have been intensively studied and proved to promote angiogenesis and prevent apoptosis in ischaemic injury. For instance, Zhang *et al*. 23 revealed that astragaloside IV, a major active constituent of Astragalus membranaceus, was a regulator of HIF‐1α/VEGF and angiogenesis through PI3K/Akt pathway in HUVECs exposed to hypoxia. Min *et al*. 24 discovered that 20(S)‐ginsenoside Rg3 prevented EC apoptosis *via* Akt‐dependent inhibition of the mitochondrial apoptotic signalling pathway. Ai *et al*. 25 illustrated that Salvia miltiorrhiza (Danshen) improved damaged cardiac angiogenesis and cardiac function induced by myocardial infarction by modulating HIF‐1α/VEGFA signalling pathway. As a compound preparation, it is difficult to tell exactly which characterized ingredients play the critical role. In the present study, we laid stress on the group effect of a compound mixture instead of a single agent. Further investigations are required to probe the characterized active pharmaceutical components that target NRG‐1/ErbB‐PI3K/Akt/mTOR signalling pathway.

It is well known that angiogenesis plays a critical role in the recovery of cardiac function in response to a number of injuries. CMECs are important participants in myocardial angiogenesis and directly supply myocardium with oxygen and nutrients to sustain metabolism. In addition, CMECs could more or less influence metabolism, contractile performance and rhythmicity of the adjacent cardiomyocytes. These effects appear to be mediated by various diffusible substances, as more recently demonstrated by NRG‐1 8, 26. When the CMEC‐derived NRG ligand binds ErbB receptors on cardiomyocyte, an endothelium–cardiomyocyte crosstalk is established through this paracrine regulating signalling. Our previous study indicated that *QL* induced NRG‐1 secretion of CMECs and protected cardiac myocytes against hypoxia‐induced apoptosis *via* ErbB4/PI3K/Akt pathway 27. As ECs contain ErbB receptors, they are also targets for autocrine signalling *via* this pathway, an important mediator of vascular preservation and angiogenic responses of endothelium. That is the reason why we focused on CMECs and NRG‐1/ErbB signalling in the present study. *QL* preserved physiological function of CMECs, characterized by improved migration ability and capillary‐like tube formation. NRG‐1/ErbB signalling was involved in this process.

Studies have reported that NRG‐1 binds ErbB receptors in isolated cardiac myocytes and regulates a variety of cellular responses, including cell survival, mitochondrial function and glucose uptake 28. In the present study, ErbB2 and ErbB4 phosphorylation was induced by NRG‐1 and then activated the PI3K/Akt/mTOR pathway in CMECs. NRG‐1 knockdown abrogated activation of Akt and mTOR, while PI3K/Akt inhibition had no effect on NRG‐1/ErbB signalling. Binding of NRG‐1 to ErbBs, which are transmembrane tyrosine kinase receptors, activates intracellular PI3K/Akt/mTOR signalling, namely a downstream pathway of NRG‐1/ErbB signalling network. As a central regulator in NRG‐1/ErbB signalling, Akt phosphorylation may contribute to CMECs functioning, including survival, growth and migration, in which mTOR serves as an important mediator 29. Studies have reported that PI3K/Akt/mTOR pathway could lead to HIF‐1α activation and regulate angiogenesis 30. *QL* has also been confirmed to attenuate progression of cardiac remodelling after ischaemia/reperfusion injury *via* mTOR activation 31. In the present study, such pro‐angiogenic effect was abolished by PI3K/Akt inhibitor, LY294002, which effectively suppressed HIF‐1α expression and beneficial effects of *QL*.

Activated HIF‐1α and its downstream VEGF confer protection against hypoxic and anoxic injury, by triggering an increase in proliferation, migration and invasion of ECs, as well as the formation of a capillary‐like tubular structure network. Hypoxia/re‐oxygenation procedure is sometimes induced to establish a model of acute hypoxic injury, and cells may adapt to this environmental stress and survive. In the present study, however, chronic anoxia was conducted with no re‐oxygenation. Under such circumstances, the protective mechanism initiated by adaptive HIF‐1α elevation seems decompensated and not sufficient, as extreme anoxia can induce apoptosis by causing hyperpermeability of the inner mitochondrial membrane and inhibiting electron transport chain 32, 33, leading to an impaired migration ability and capillary‐like tube formation of CMECs. Fortunately, our herbal remedy up‐regulated HIF‐1α and VEGF expressions, which directly facilitated adaptation and survival of ECs from normoxia to anoxia.

PPARγ activation could reduce vascular injury stimulated by ox‐LDL 34. In the current setting of anoxic injury, however, it did not mediate the protective effects of *QL*. Although recent study has argued the role of PPARγ in *QL*‐induced attenuation of cardiac remodelling after acute myocardial injury 15, other cell types (*i.e*. cardiomyocyte) rather than CMECs may action.

It has been reported that PI3K/Akt signal transduction provides cells with a survival signal that allows them to withstand apoptotic stimuli, whose targets downstream involves FoxO subfamily of Forkhead transcription factors, Bcl‐2 family, NF‐κB and cAMP‐responsive element binding protein (CREB) pathways, etc. 35. Our results suggested that the NRG‐1/ErbB and PI3K/Akt/mTOR signalling mediated the anti‐apoptotic activity of *QL* on CMECs, although the concrete mechanism remained to be investigated in further study.

In conclusion, the present study revealed the beneficial effects of *QL* on CMECs in response to anoxia in terms of promoting cell migration, neovascularization and reducing apoptosis. These protective effects might be mediated by an autocrine effect of NRG‐1/ErbB signalling *via* activation of PI3K/Akt/mTOR pathway. HIF‐1α and VEGF also contribute to angiogenesis in this setting, although the exact anti‐apoptotic mechanism warrants further investigation. Our results shed some light on the potential importance of endothelium‐derived NRG‐1 in angiogenic and pro‐survival response to anoxia and provided new insights of therapeutic approach to CMEC anoxia.

## Conflict of interest statement

The authors declare that there are no conflicts of interest.

## Supporting information


**Figure S1** Isolation and identification of CMECs, screening for optimal anoxia time, and NRG‐1 siRNA transfection validation with five independent experiments performed.
