# Supplementary figures and images for: Qiliqiangxin protects against anoxic injury in cardiac microvascular endothelial cells via NRG‐1/ErbB‐PI3K/Akt/mTOR pathway
Source: J Cell Mol Med. 2017 Mar 8;21(9):1905–14. doi: 10.1111/jcmm.13111 (PMC5571527; doi:10.1111/jcmm.13111)

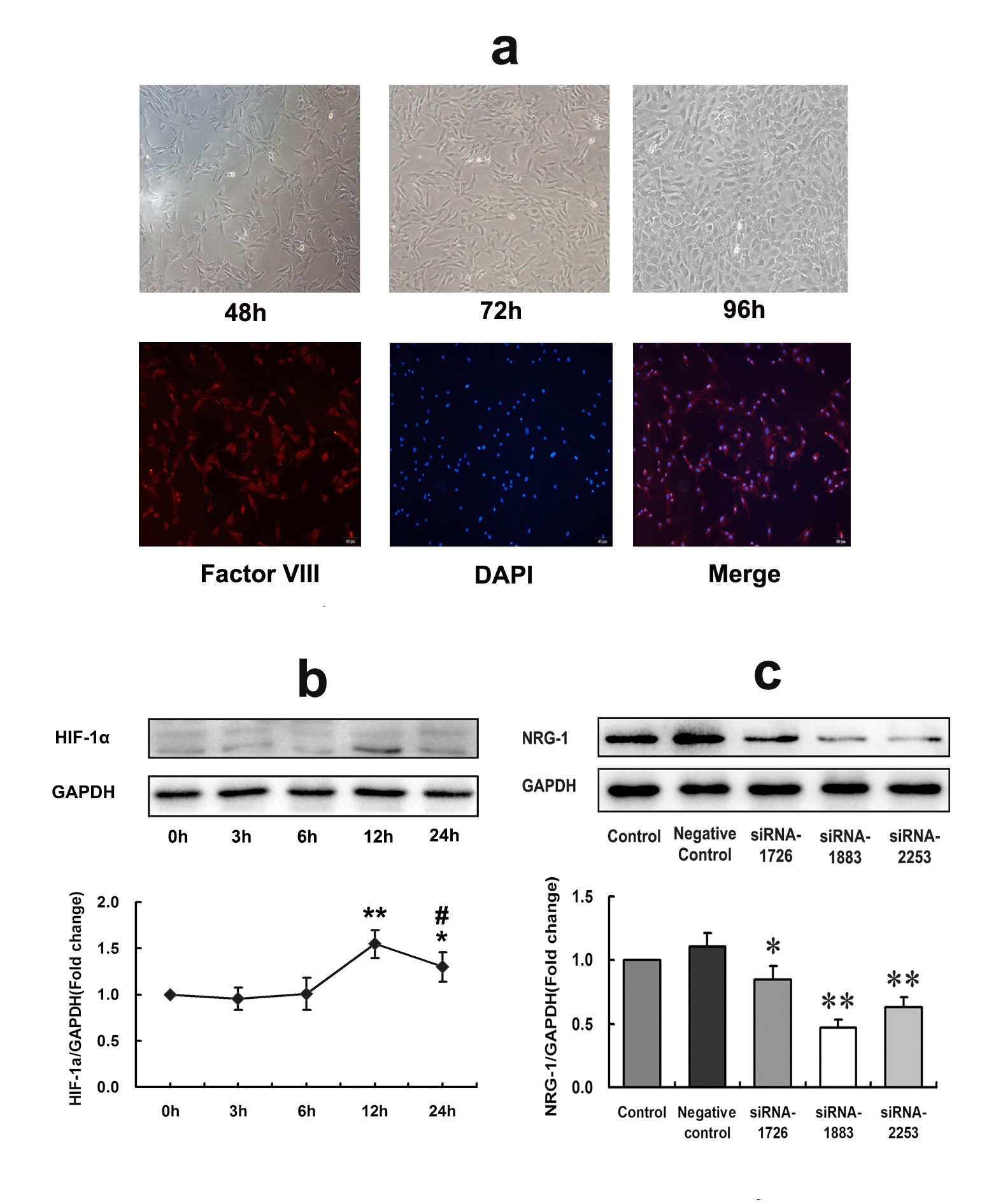

Supplement: Supplementary file 1 — Figure S1 Isolation and identification of CMECs, screening for optimal anoxia time, and NRG‐1 siRNA transfection validation with five independent experiments performed. [file JCMM-21-1905-s001.tif]
